# Healthcare provider and pregnant women’s perspectives on the implementation of intermittent screening and treatment with dihydroartemisinin–piperaquine for malaria in pregnancy in western Kenya: a qualitative study

**DOI:** 10.1186/s12936-021-03826-8

**Published:** 2021-06-29

**Authors:** Jenna Hoyt, Jenny Hill, Florence Achieng, Peter Ouma, Simon Kariuki, Meghna Desai, Jayne Webster

**Affiliations:** 1 Department of Clinical Sciences, Liverpool School of Tropical Medicine, Liverpool, UK; 2 Kenya Medical Research Institute/Centre for Global Health Research, Kisumu, Kenya; 3 Centers for Disease Control and Prevention, Atlanta, GA USA; 4 Disease Control Department, London School of Tropical Medicine and Hygiene, London, UK

**Keywords:** Feasibility, Intermittent screening and treatment, Intermittent preventive treatment, Dihydroartemisinin–piperaquine, Health system delivery, Pregnant women, Healthcare providers, Kenya

## Abstract

**Background:**

In malaria endemic regions in Kenya, pregnant women are offered long-lasting insecticidal nets and intermittent preventive treatment (IPTp) with sulfadoxine–pyrimethamine (SP) at antenatal care (ANC) to prevent the adverse effects of malaria. Fears of growing SP resistance have heightened the search for alternative strategies. The implementation feasibility of intermittent screening and treatment (ISTp) with dihydroartemisinin–piperaquine (DP) in routine ANC settings was evaluated using qualitative and quantitative methods, including the exploration of healthcare provider and pregnant women’s perceptions.

**Methods:**

Qualitative methods included data from 13 focus group discussions (FGDs) with pregnant women and 43 in-depth interviews with healthcare providers delivering ANC services. FGDs were conducted with women who had received either ISTp-DP or current policy (IPTp-SP). Thematic analysis was used to explore experiences among women and providers and findings were used to provide insights into results of the parallel quantitative study.

**Results:**

Women were accepting of testing with rapid diagnostic tests (RDTs) and receiving treatment if malaria positive. Providers perceived DP to be an effective drug and well tolerated by women. Some providers indicated a preference for test and treat strategies to reduce unnecessary exposure to medication in pregnancy, others preferred a hybrid strategy combining screening at every ANC visit followed by IPTp-SP for women who tested negative, due to the perception that RDTs missed some infections and concerns about the growing resistance to SP. Testing with RDTs during ANC was appreciated as it was perceived to reduce wait times. The positive attitude of healthcare providers towards ISTp supports findings from the quantitative study that showed a high proportion (90%) of women were tested at ANC. There were concerns about affordability of DP and the availability of sufficient RDT stocks.

**Conclusion:**

In ANC settings, healthcare providers and pregnant women found ISTp-DP to be an acceptable strategy for preventing malaria in pregnancy when compared with IPTp-SP. DP was considered an effective anti-malarial and a suitable alternative to IPTp-SP in the context of SP resistance. Despite providers’ lack of confidence in RDT results at current levels of sensitivity and specificity, the quantitative findings show their willingness to test women routinely at ANC.

**Supplementary Information:**

The online version contains supplementary material available at 10.1186/s12936-021-03826-8.

## Background

Malaria in pregnancy (MiP) is associated with poor health outcomes for both mother and baby, with a high number of low birthweight deliveries attributed to MiP [1]. The World Health Organization (WHO) currently recommends intermittent preventive treatment with sulfadoxine–pyrimethamine (IPTp-SP), use of long-lasting insecticidal nets (LLINs) and effective case management as interventions to reduce MiP and they are primarily delivered through the antenatal care (ANC) platform. However, against the backdrop of declining transmission rates across much of Kenya and the growing threat of SP resistance, the Division of National Malaria Programme (DNMP) has recently shifted the policy for control of MiP from IPTp-SP to passive case detection alongside the continued use of LLINs in all but three endemic provinces [2]. Where IPTp-SP remains policy, concerns have been raised regarding the possible increased risk of fetal anaemia in areas with high levels of SP resistance [3], although more recent studies indicate that SP continues to be effective in reducing low birth weight outcomes in areas of high resistance but is compromised in areas with highly resistant parasites [4]. Nevertheless, these concerns have accelerated the search for alternative drugs for IPTp or new strategies that are at least as effective in controlling MiP and can be implemented and delivered through routine ANC services.

One potential alternative strategy is intermittent screening and treatment (ISTp) for malaria in pregnancy. ISTp involves testing pregnant women at every ANC visit using microscopy or rapid diagnostic tests (RDTs) and providing treatment only to those who test positive, with women who test negative receiving no anti-malarials. ISTp has now been tested in a number of randomized controlled trials (RCTs) in East and West Africa [5, 6], Indonesia [7] and India [8] together with an evaluation of implementation in the routine setting [9] and in studies of user and provider acceptability in some countries [10–13]. The ISTp strategy was explored previously in western Kenya under trial conditions and was found to be broadly acceptable to both pregnant women and healthcare providers [12], but this will be the first study to examine perceptions and experiences of implementation in the routine setting in this region.

To complement a large RCT comparing ISTp or IPTp with DP against the current policy of IPTp with SP, an evaluation was conducted to assess the implementation feasibility of one of the trial interventions, ISTp-DP in health facilities outside of the trial setting, compared to implementation of the national policy, IPTp-SP. The evaluation included quantitative and qualitative components, the qualitative component reported on here was carried out to explore the attitudes of healthcare providers and pregnant women towards key elements of the ISTp strategy and assess possible challenges for implementing ISTp under routine conditions. Findings from this study are used to provide insights into some of the key results from the parallel quantitative study.

## Methods

### Study site and context

The study was conducted in four sub-counties in Kisumu County in western Kenya—Muhoroni, Nyakach, Kisumu West, and Seme. At the time of the study, the population of Kisumu County was approximately 970,000 [14]. Malaria transmission in this region is stable and perennial. MiP guidelines for malaria endemic regions in Kenya include the use of LLINs (distributed through routine ANC channels, social marketing and mass campaigns), prompt diagnosis and case management of active infections, and IPTp with SP delivered via directly observed therapy (DOT) at every ANC visit starting in the 2^nd^ trimester and given at least 4 weeks apart with a target of at least 3 doses throughout the pregnancy [2, 15]. ANC coverage is high in the lake endemic zone, which includes Kisumu county, with 97.3% of pregnant women receiving ANC care from a skilled provider [16]. In 2015, 54.7% of pregnant women in the endemic lake regions received 2 or more doses of SP, however only 35.3% received the recommended 3 or more doses of SP in their last pregnancy [16].

### Intervention

Full details of the intervention implementation are reported in the companion paper Hill et al*.* [17]. In summary, the implementation feasibility study was conducted in twelve health facilities that were not involved in the RCT. For each strategy (IPTp-SP or ISTp-DP) six health facilities were selected. Health facility selection was based on (1) type of government owned facility—level 4 (district and sub-district hospitals) or level 3 (health centres) and (2) the feasibility of achieving the required sample size of pregnant women attending ANC. Due to resource constraints, in-depth interviews (IDIs) with healthcare providers and focus group discussions (FGDs) with pregnant women were carried out in a subset of the implementation facilities. Participants were selected from four of the six intervention health facilities (ISTp-DP) and four of the six control (IPTp-SP) health facilities. The 8 out of 12 health facilities were selected based on logistic and resource feasibility whilst ensuring inclusion of a mix of districts and level 4 and level 3 health facilities.

Pregnant women attending the selected health facilities received the usual focused ANC package and, depending on the study arm, either: (1) IPTp with a single dose of SP delivered by ANC staff via directly observed therapy (DOT), or (2) ISTp with DP for which women were tested using RDTs and if positive, provided with a first dose of DP by DOT and instructions to complete the 2nd and 3rd dose of DP at home (the DP doses are taken once per day for 3 days). Women who tested RDT negative were given no anti-malarial medication. The health facilities were provided with sufficient RDTs [CareStart Malaria HRP2 (Pf); AccessBio Inc, USA], DP, and SP for the study period. The RDTs provided were the same as those procured and distributed by the DNMP.

### Evaluation design

The evaluation employed a mixed methods study design, and this paper reports on the qualitative findings. Methods used in the qualitative evaluation comprised IDIs with healthcare providers and FGDs with pregnant women in each study arm. The aim was to understand perceptions of both providers and pregnant women towards the intervention (IST-DP), specifically examining women’s acceptability of the core components of the interventions i.e. blood tests and adherence to drugs. The findings of the qualitative study were then used to interpret some of the key results of the quantitative study particularly across components of the feasibility framework defined by Bowen et al*.* [18].

### Participants and study procedures

The IDIs and FGDs were conducted between February and June 2015, six months after the pilot implementation of ISTp. IDIs were conducted with healthcare providers in four of the intervention (ISTp-DP) and four of the control (IPTp-SP) health facilities, together with County health management team members in all four districts. FGDs were conducted with pregnant women who had attended ANC at either the intervention or control health facilities.

### In-depth interviews with providers

Healthcare providers were purposively selected to include those involved with different elements of the intervention and control strategies, including health facility managers, ANC nurses, laboratory technicians and pharmacists, and County health management team members. Individual interviews were conducted with healthcare providers in English, using a semi-structured interview guide (Additional file 1). Interview themes included: (1) perceptions of the ISTp-DP strategy vs the current policy of IPTp-SP; (2) workplace adaptations that would be required if ISTp-DP were adopted, (3) recommendations to ensure effective implementation of ISTp-DP, (4) perceptions of service delivery challenges and opportunities with ISTp-DP (including use of RDTs in the ANC). Interviews were digitally recorded and transcribed verbatim.

### FGDs with pregnant women

FGDs were undertaken with women who had attended ANC in the implementation feasibility study control or intervention health facilities. Four categories of FGD were conducted including groups comprising women who had received either: (1) IPTp-SP; (2) ISTp-DP and were RDT negative; (3) ISTp-DP and were RDT positive at least once; or (4) either IPTp-SP or ISTp-DP—a mixed heterogenous group. The FGDs with pregnant women followed a topic guide to elucidate women’s perspectives and experiences with regards to ANC services, blood testing (finger pricks with RDTs), taking of medication during pregnancy (including concerns about taking SP or DP during pregnancy) and motivation for attending ANC (Additional file 2). Interviews were conducted in Luo and Swahili and digitally recorded, then transcribed and translated into English.

### Data analysis

The transcripts from both IDIs and FGDs were transferred to NVivo 10 for coding and analysis. Data from the IDIs and FGDs were coded separately by one investigator and disagreements in the coding and analysis were discussed among the research team until a consensus was reached. The framework for analysis for healthcare provider IDIs included and expanded upon two published frameworks including: (1) health systems building blocks (finance, governance, health information, human resources, products and technology, service delivery) [19], (2) feasibility framework (acceptability, demand, implementation, practicality, integration, adaptation, expansion) [18], and additionally included (3) provider perceptions of pregnant women’s acceptance of intervention components: DP vs. SP, blood testing, and ISTp-DP vs. IPTp-SP. Data could be coded multiple times to fit themes in each of the different components of the framework to enable synthesized and individual analysis of the data.

A framework for analysis for the FGDs with pregnant women was developed comprising: (1) access framework (availability, accessibility, accommodation, affordability, acceptability) [20], (2) perceptions of blood testing, (3) adherence to drugs (DP and SP), and (4) perception of ANC services. Here, acceptability is defined as ‘the extent to which an intervention (blood testing and taking DP) is judged as suitable, satisfying or attractive’ [20]. For both IDIs and FGDs thematic analysis was used and emergent themes and sub-themes were inductively added to the deductive codes within the frameworks. Quotes were extracted to add richness and support the analysis. Quote labels use participant identification numbers and provider role to protect anonymity.

## Results

A total of 43 IDIs with healthcare providers were conducted across eight health facilities in the four sub-counties (Kisumu West, Muhuroni, Nyakach and Seme); five of the facilities were Level 4 and three were Level 3 facilities. The healthcare providers interviewed included: 16 ANC nurses, 7 in-charges, 8 laboratory technicians, 7 pharmacists, and 5 members of the County health management team (Table 1). FGDs were conducted with a total of 95 women attending 13 FGDs across the control and intervention groups (Table 2). The results presented here are the themes and sub-themes from both healthcare provider and pregnant women’s perspectives mapped, where applicable, across the seven feasibility constructs (Table 3).

**Table 1:** Healthcare providers interviewed by cadre and site

Healthcare provider cadre	Control sites, (IPTp SP)	Intervention sites, (ISTp DP)	Total interviewed
ANC nurse	7	9	16
Facility managers	3	4	7
Laboratory technician	4	4	8
Pharmacist	4	3	7
County health management team	1	4	5
Total	19	24	43

*ANC* antenatal care, *ISTp* intermittent screening and treatment, *IPTp* intermittent preventive treatment, *DP* dihydroartemisinin–piperaquine, *SP* sulfadoxine–pyrimethamine

**Table 2:** Focus group discussions with pregnant women by intervention arm

FGD groups	Number of FGDs	Total number of women
IPTp-SP	4	27
ISTp—negative	3	20
ISTp—positive	3	25
ISTp—heterogeneous	3	23
Total	13	95

Negative: RDT negative women, positive: RDT positive women, heterogeneous: IPTp & ISTp

*FGD* focus group discussion

**Table 3:** Experiences and perceptions on the feasibility of ISTp-DP vs. IPTp-SP among healthcare providers and pregnant women

Feasibility framework	Themes	Pregnant women	Healthcare providers
		Sub-themes	Sub-themes
Acceptability	ISTp vs. IPTp	Women want to know if they have an illness and either receive prevention or treatment; happy to be tested, gives peace of mind; testing can be painful; women not always told what the test is for	ISTp: better to test before treating for malaria; reduce unnecessary medication in pregnancy; good to identify asymptomatic cases; prefer not to rely on RDTs; women may not want repeated testing; complaints of testing at every visit^a^ IPTp: good because focused on prevention; concerns about resistance to SP; women accepted IPTp once benefits were explained^a^ IPT/IST: Women are accepting of tests and treatments offered at ANC^a^
	DP and SP	Women are not always sure about taking drugs during pregnancy; complaints about side effects nausea, dizziness, headaches (both SP and DP but more frequent with SP); women happy with DP, it cured malaria; not happy to take SP on empty stomach	n/a
	RDTs vs. microscopy	n/a	RDTs: fast, convenient and easy to use; good alternative to microscopy when no laboratory technician or electricity; not always accurate; they do not detect all species of malaria; some confusion that finger prick was for HIV testing^a^; RDTs more accepted because of HIV testing^a^
Demand (pregnant women) Implementation/integration (Healthcare providers)	ISTp delivery at ANC	Women are motivated to return to ANC to be tested for malaria	“One-stop-shop” is good for women, reduces wait times, continuity of care; better follow up for women receiving treatment; better to dispense anti-malarials at ANC; only pharmacy should dispense drugs; need adequate staffing to carry out screening at ANC
Demand	DP vs. SP	Women are motivated to attend ANC to receive drugs, treatments	DP: effective drug as women don’t return with malaria; has shorter dosing regimen than quinine; expensive for women to buy if out of stock at health facility; should only be used for treatment, don’t want to build resistance to it SP: has been good in reducing the burden of MiP; concerns about resistance; good for prevention because can be given in one dose by DOT
Practicality	Sustainability of RDTs and DP	n/a	Stock outs for both RDTs and DP; women may not afford DP if they need to buy from private providers; limited as facilities can only order from KEMSA
Adaptation/expansion	ISTp delivery at ANC	n/a	Have high workloads already, RDTs in ANC may add to that; RDTs at ANC save time as we don’t need to wait from women to return from the laboratory; reduced workload for laboratory staff; happy for ANC providers to administer RDTs if properly trained

*ISTp* intermittent screening and treatment, *IPTp* intermittent preventive treatment, *DP* dihydroartemisinin–piperaquine, *SP* sulfadoxine–pyrimethamine, *RDT* rapid diagnostic test, *ANC* antenatal care, *KEMSA* Kenya Medical Supplies Authority

^a^Health care provider perceptions of pregnant women

### Healthcare provider perspectives

**Fig. 1:**
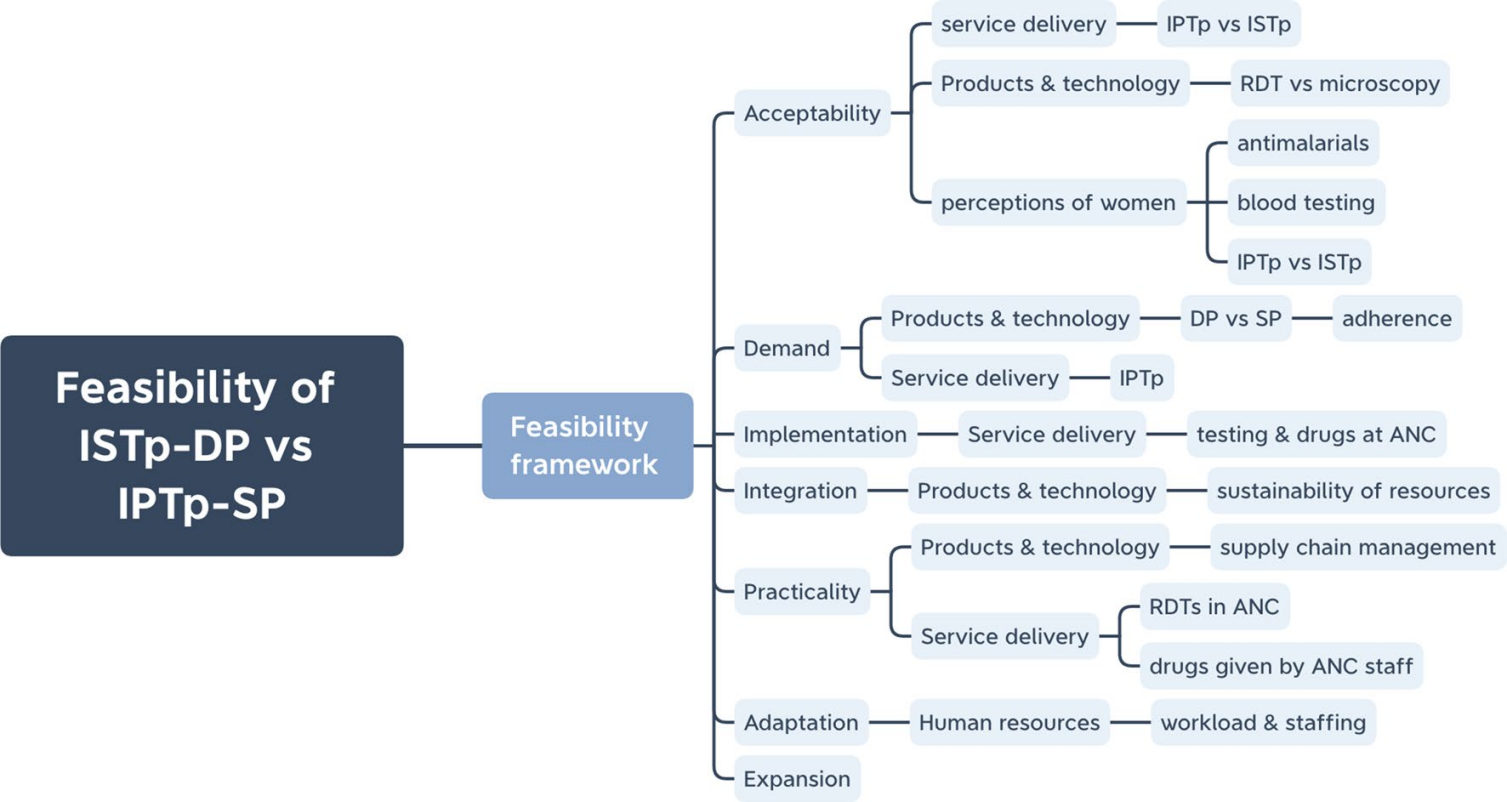
Healthcare provider perceptions across feasibility constructs [18] and health systems building blocks [19]

Healthcare provider perceptions are explored across the feasibility constructs, specifically understanding acceptability towards key components of the intervention as well as how ISTp fits into routine ANC services and potential implementation challenges should the strategy be adopted. Figure 1 illustrates how the health system building blocks relate to the different constructs of the feasibility framework and indicate what elements of the health system need to be considered for implementation, in particular, products and technology, service delivery and human resources.

### Acceptability—to what extent do healthcare providers accept ISTp or IPTp?

In general, healthcare providers responded positively towards ISTp as a MiP control strategy. Across various provider cadres ISTp was described as beneficial because women would be tested before receiving treatment, thereby reducing potentially unnecessary medication during pregnancy. In addition, some providers reported that women complained about having to take drugs when they felt fine and preferred taking drugs after testing positive. Many providers felt that regular testing at ANC would help identify active cases (women with malaria parasitaemia) as MiP is often asymptomatic.

*“Interviewer[I]: How would you feel about every pregnant woman being given an RDT test at every ANC visit and if they were positive for malaria, they would be given an anti-malarial drug, if they were negative, they would not receive any anti-malarial? Respondent [R]: I feel it is okay. It is the best approach I think…because we have actually seen that she is not having malaria so you don’t give the drug but if she is positive you treat her then she goes home you* [know] *she is going to be safe for the whole month before she comes back again.”* IDI-14, ANC nurse

*“I: How about the community perception of pregnant women, what do they say? R: I think they are welcoming the study because what they are saying…ok that last time they were being given the SPs without the tests but right now they are given drugs only when they test positive.”* IDI-10, ANC nurse

Contrary views by providers focused on the use of RDTs for testing and the limitations with regards to accuracy. Some participants, mostly laboratory technicians, liked ISTp as a strategy but felt it should not be implemented if it meant the use of RDTs instead of microscopy. Reliability of RDT stocks and pregnant woman confusion over repeated testing were also mentioned as possible challenges to implementation. Providers perceived the main benefits of the current IPTp strategy to be that it was prevention focused and did not rely on testing. Concerns about growing resistance to SP were considered a downside to the current policy. Several providers described a hybrid ISTp/IPTp strategy where women could be tested at every ANC visit and treated if positive for malaria but if negative, they would still receive a prophylactic dose of SP.

*“I: …So how would you feel about every pregnant woman being given an RDT test at every ANC visit? R: It’s good, because there are people who have malaria but they don’t show symptoms. And in pregnancy, malaria can easily cross placenta barrier and infect the foetus. So I think it’s good I: So when they test positive what happens? R: They are treated, we use Duocotexin [dihydroartemisinin–piperaquine] right now I: How about those who turn negative? R: Those who turn negative, they could be given prophylaxis.”* IDI-27, In-charge

#### RDTs versus microscopy

Overall, ANC providers reacted favourably towards RDT use for malaria testing acknowledging that they were easy to use, fast and convenient. Many participants reported that RDTs were effective alternatives to microscopy in resource-limited settings, during power outages and when laboratory technicians were not available. However, the limitations of RDTs were also described with providers reporting issues with accuracy and concern over the devices’ inability to detect certain malaria species. Laboratory technicians, pharmacists and facility managers were more vocal about the challenges of using RDTs whereas the benefits of performing RDTs in ANC were most often reported by ANC nurses.

*“I: …So what do you think about the use of RDTs for diagnosis of malaria as an alternative to blood slides? R: It’s efficient, it’s time saving and it’s appropriate.”* IDI-3, ANC nurse

*“I: What do you think about the use of RDT for diagnosis of malaria as an alternative to blood slide R: I really don’t know if RDT is 100% because I hear RDT will only pick specific parasites of malaria not all, so at one point it might give you a false reading if a patient is positive yet it gives negative since it has not picked the malarial parasites that are there in the blood*.” IDI-37, Pharmacist

### Demand—to what extent is DP perceived to have positive effects, to be used, and demanded by pregnant women compared to SP?

Healthcare providers at intervention sites responded positively towards the use of DP for treatment of MiP, finding it effective and noting that women don’t return with malaria if they have taken it.

*“I: So what do you think about them, like let’s talk of DHA*–*piperaquine [DP]. You have used it. R: According to my experience it works immediately because after giving the mothers next visit you are testing them, they don’t have malaria, in fact they respond with the drugs, it works well.”* IDI-11, ANC nurse

Other reported benefits of DP included a shorter dosing regimen compared with quinine and mild or non-existent side effects. It was also suggested that DP might be preferable to women as it was a new drug. The main concern with DP was that should the ANC be out of stock, it would be costly for women to have to buy it out of pocket. A facility in-charge felt that DP should not be used for prevention, only treatment, to avoid building resistance to this new drug.

Although providers acknowledged that SP had helped reduce the burden of MiP there was apprehension about growing resistance alongside reports of women returning with malaria despite having taken SP. However, SP was preferable for use as IPTp because it required only a single dose that could be given by DOT, thereby eliminating compliance issues.

#### Adherence to drugs

There was a clear consensus among providers that longer dosing regimens lead to poor adherence and, as such, artemether–lumefantrine (AL) and DP were preferred to oral quinine. Providers also felt that drugs with side effects reduced adherence, again with some providers expressing a preference for DP over quinine due to the perception that women experienced fewer side effects with DP. Providers voiced their concerns about lack of adherence because they felt that women who did not adhere to the full course usually returned to the health facility due to relapse or complications.

*“I: So what are the implications for adherence to full dose, or future prescriptions? R: If they don’t comply with the treatment and complete, they may come back with malaria still which has not resolved, and most of the time, it ends up with treatment failure. Poor adherence, there is treatment failure.”* IDI-27, In-charge

### Implementation—to what extent is ISTp being carried out in health facilities, and where?

Healthcare providers discussed the benefits of a “one-stop-shop” at ANC where women can be tested and receive treatment without needing to go to the laboratory or pharmacy. The benefits were described as reduced waiting times and better follow up for treatment, as the first dose would be administered by DOT. Several providers mentioned that receiving all services at ANC would encourage women to attend as waiting times would be reduced.

*“I: How would you feel if the pregnant mother attending ANC who tested positive for malaria were given their anti-malaria treatment by the ANC staff? And/or the ANC staff also implemented the RDTs? R: That one is very much okay it will not waste time for this ante natal mothers to go and line up again at the laboratory then you come here you line up go to the pharmacy you line up. These people are people who need support and rest so if we save for them time I think is okay*.” IDI-13, ANC nurse

### Integration—to what extent has ISTp been integrated into the existing system?

Overall ANC providers said they would prefer to dispense the anti-malarials at ANC, however some felt that medications should be dispensed by the pharmacy and that it was only possible as long as there was sufficient staff to manage it. Pharmacists preferred anti-malarials to be dispensed by them as it was in their job description and they had the proper training. Similarly, there was strong support by ANC nurses to carry out malaria testing with RDTs in ANC but again there were concerns about sufficient staffing and increases in workload, although primarily voiced by providers at the control sites. Laboratory technicians were supportive but felt that they should still oversee the testing for reporting and control purposes. A laboratory technician pointed out that women would still need to go to the laboratory for routine screening.

*“R:…we would not like to give them to the ANC staffs…those are expensive drugs. I: But what’s the problem if an ANC staff gives these drugs or treatment? R: There is no problem about them giving drugs. The issue is about management of commodities, for you to be aware of what you are doing, what you are using, what you have available and maybe the drugs are going to the right person and not anybody else. You see we can’t have several people dispensing at the facility.”* IDI-40, Pharmacist

### Practicality—to what extent is ISTp being carried out using existing resources?

Concerns about the availability of RDTs and DP were expressed by providers, as stock outs of both were reported. A further issue with regards to DP was the high cost of the drug and whether or not that would affect supply. During stock outs women are often asked to purchase drugs from outside the facility and some providers doubted whether women would be able to afford DP. Kenya Medical Supplies Authority (KEMSA) is the national supplier of drugs and RDTs, which limited the procurement options when stockouts occurred. Several providers mentioned trying to source drugs and RDTs from other facilities when they ran out.

### Adaptation—to what extent have changes been made to existing systems to implement ISTp? Expansion—to what extent can ISTp be scaled up and expanded?

Healthcare providers at control sites reacted positively to the use of RDTs in the ANC to test pregnant women but only if staffing levels were sufficient. Other providers felt that testing pregnant women at ANC would save time as they would not have to wait for laboratory results. Laboratory technicians supported the idea of ANC providers testing women with RDTs but only if they continued to oversee reporting and monitoring. There was broad support for ANC providers to carry out testing with RDTs as long as they received appropriate training.

### Pregnant women perspectives

**Fig. 2:**
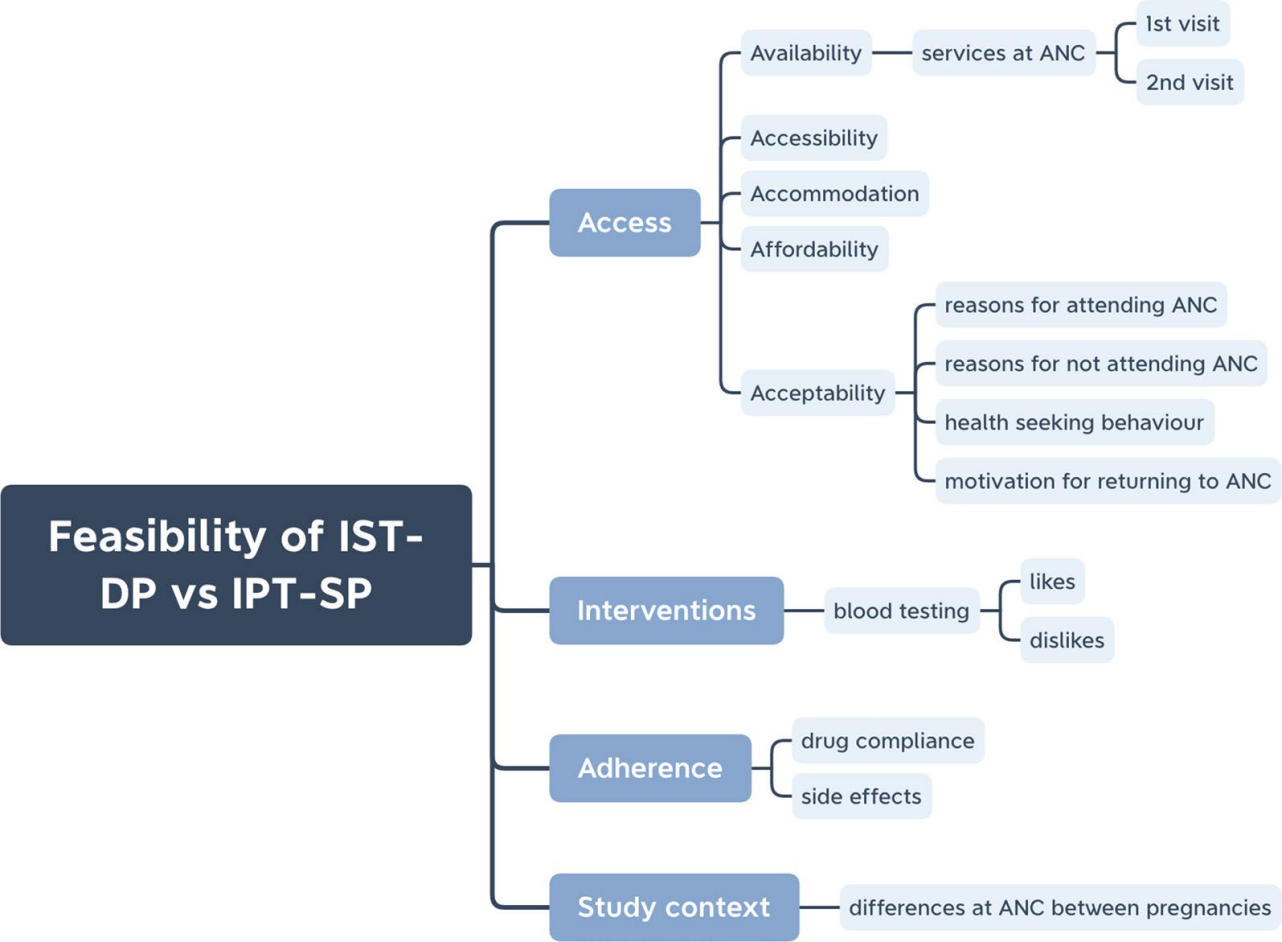
Analysis framework for perceptions of pregnant women

The perceptions of pregnant women towards key components of the ISTp strategy are predominantly captured within the acceptability construct of the access framework, which aligns with the feasibility framework. Additional themes around blood testing and adherence fit within the feasibility constructs of acceptability and demand (Fig. 2). Specifically, women’s perceptions capture to what extent they are accepting of both malaria testing and drugs (DP) and motivated to attend ANC for those interventions.

### Acceptability—to what extent are components of ISTp judged as suitable, satisfying, or attractive?

#### Anti-malarial drugs and testing

Pregnant women across the groups responded favourably to being tested for malaria and receiving treatment if positive. It also provided peace of mind to those who tested negative to know they did not have malaria. Generally, women wanted to know if they had any diseases or conditions that might compromise their health or that of their baby. ‘Knowing my blood level’, which refers to haemoglobin levels and whether or not they are anaemic, was frequently reported by women across all groups. Negative views on testing were that it was painful, a lot of blood was taken and sometimes the reason for the test was not explained.

*“R2: I did not like it because even if you ask them what test they are doing they do not tell you and also even after they give you the result they are not even ready to tell you what it is and so you do not know the advantage or disadvantage.”* FGD 8, IST negative

However, women were clearly willing to accept the pain and discomfort of finger prick tests for the benefits of being tested for malaria and receiving treatment.

*“R4: What I did not like is that the injection was very painful and what I liked is that I knew my status and again I was happy that I had two children and I was going to give birth to them with the same status. So I liked that.*” FGD 13, IST positive

When asked about their experiences taking anti-malarials, pregnant women from the control group (IPTp-SP) shared mixed opinions. Some women were happy, as they did not contract malaria during their pregnancy, while others were not happy to have to take SP on an empty stomach and feared a reaction. One woman reported that she still got malaria despite taking SP. Women from the intervention group (ISTp-DP) reported being satisfied with DP, as it was effective and cured them of malaria.

*“I: What can you say about the drugs that you were given? R4: I can say that they were good. Ever since I finished the dose, I haven’t seen any serious signs of any disease in me. So I can say that they were good*.” FGD 13, IST positive

Women in both groups reported side effects that they believed were caused by the anti-malarial drugs, which included dizziness, nausea and headaches for both SP and DP. Reported side effects were mentioned more often among women in the SP groups.

#### Adherence to drugs

Women reported being given a number of different drugs at ANC and described them largely by colour or function. Most women reported receiving “blood boosters” (iron tablets), following the dosing instructions and completing the regimen at home. A few women described not being able to finish as they delivered the baby before they finished the tablets or that they increased their appetite too much. One woman from an IPTp group reported receiving quinine tablets, but not finishing them because of unpleasant side effects.

*“R5: I had fever sometimes during this pregnancy and I came to the hospital and was given thirty tablets of quinine. I did not finish the dose because whenever I took those drugs, I could feel dizzy and I get nauseated and sometimes I get itchy on the skin and my ears were blocked. So, I could not continue with them.”* FGD 3, IPTp

### Demand—to what extent is ISTp likely to be used (how much demand is likely to exist)?

#### ANC attendance

Women in both intervention groups were motivated to attend ANC to know their status (with regards to HIV) and to look for other diseases, which included getting tested for malaria and receiving treatment if needed or to prevent malaria.

*“R1: I wanted to know my status on malaria because you know pregnant women are prone to malaria. So I wanted to know whether I also had malaria and also the condition of the child. Whether the child was moving or if it was not moving.”* FGD 11, ISTp HET

Other important reasons for attending ANC included to check the health and position of the baby and receive drugs. Women from both intervention groups reported that their main reason for returning to ANC was because they felt an improvement in their health after receiving services or treatment at a previous ANC visit. Among women who received ISTp, malaria testing was a primary reason for returning to ANC. Interestingly, women reported that the main reason other women do not attend ANC is because they fear testing (primarily for HIV).

*“I: Okay and why is it that many women do not go for the antenatal clinics? R6: Some women do not go to the clinic because they think that they can be tested and be found positive and so they are so afraid. I: Positive in what. R6: For the HIV test.”* FGD 2, IPTp

Other reasons reported by women for not attending ANC included lack of education and awareness, preference for/influence of traditional birth attendants (TBAs) and the distance to reach facilities.

#### Health-seeking behaviour

When asked what symptoms prompted them to seek care pregnant women most commonly reported ‘headaches’, ‘feeling tired’, ‘nausea', and 'vomiting’. ‘Suspecting malaria’ and ‘loss of appetite’ were also reported. Of the women who mentioned having experienced a fever in pregnancy, most of them sought treatment at the hospital. Some women reported other health-seeking behaviour, such as self-medicating from a local kiosk or pharmacy. A few women reported that they waited until their next ANC appointment and then informed the healthcare provider. One woman said she did not seek treatment because you cannot take drugs while pregnant.

*“R4: What can make someone to leave the house to go to the hospital is when sometimes you feel so tired or you have some abdominal pains. Maybe you feel you are tired like you have malaria. You know you cannot just go to the shop and buy drugs; you will have to go to the hospital so that you are tested. Maybe you feel pain in the lower abdomen. You will have to come to the hospital so that you are checked and given the correct drug for the problem.”* FGD 6, ISTp positive

### Integration of qualitative and quantitative findings

Each of these strategies is composed of several delivery steps, however the ISTp-DP strategy has more steps and is more complex to deliver. Healthcare providers were supportive of the test and treat (ISTp) strategy for MiP and appreciated that administering RDTs at ANC would improve the continuity of care for pregnant women. This was reflected in the findings from the quantitative study that shows a high proportion (90%) of eligible women attending ANC were tested with an RDT and 71% of women that tested positive were given the correct dose of DP [17]. There were however issues with healthcare provider adherence to other components of the ISTp strategy, with only 31% of pregnant women who were given DP receiving the first dose by DOT and only 6% of women reported being given instructions by the provider on how to take the remaining doses. Healthcare providers discussed the challenges with adherence to multiday treatment regimens that produced side effects (such as with quinine), and many felt that the shorter regimen of DP and relatively few associated side effects meant it may be better tolerated by women. Providers also shared their perception that women prefer to take drugs when used for treatment rather than for prevention and felt confident women would take the doses as required. Some providers indicated that women reportedly did not like to take drugs if they were not ill and welcomed treatment only if they tested positive. This view was confirmed by pregnant women who reported being motivated to attend ANC to get tested to know if they have malaria and receive treatment if positive. Provider adherence to DOT was higher among women receiving standard care of IPTp with SP (57%) [17]. That provider adherence to DOT was higher when delivering IPTp-SP than ISTp-DP aligns with the perception of providers that women are less motivated to take drugs, such as SP when they were not ill.

## Discussion

This is the first published study on healthcare provider and pregnant women perspectives regarding the feasibility of implementing ISTp-DP in an operational setting in sub-Saharan Africa. The concept of providing treatment after a positive test result was widely welcomed by healthcare providers, who highlighted the reduction of unnecessary exposure to anti-malarials during pregnancy and the growing threat of resistance to SP used for IPTp. Both strategies were seen to deal with the asymptomatic nature of MiP but some providers thought preventive treatment was the best way to ensure women were protected, while others preferred regular screening. There was interest in a hybrid strategy that provided IPTp-SP to women who tested RDT negative.

Pregnant women were accepting of finger prick tests for malaria and reacted positively towards DP as a treatment option. Women were motivated to attend ANC to detect or prevent illness and look after the health of their baby, which included undergoing diagnostic tests and receiving drugs, accepting both as part of the ANC package. Women in both groups were generally accepting of ISTp and IPTp suggesting that they were less concerned with specific interventions and more focused on the overall health benefits of ANC, a finding also described in a study of pregnant women’s acceptability of ISTp versus IPTp from Ghana [21] and supported by the findings in the acceptability trial [12]. Despite this general acceptability by both healthcare providers and pregnant women towards ISTp, there remain legitimate concerns with regards to key elements of the strategy and how those challenges may affect implementation and scale up, in particular ANC provider performance, such as delivering DP by DOT. Should ISTp become policy, intervention studies will be needed to explore how these challenges can be overcome.

The low sensitivity and specificity of the available RDTs was seen as a major stumbling block to the implementation and scale-up of ISTp. Concerns about only detecting *P. falciparum* species were raised along with reports of false positives if women had recently had malaria and been treated. These challenges were most often described by laboratory technicians, pharmacists and facility in-charges, whereas front line ANC providers focused on the benefits of using RDTs, enabling fast, easy testing of pregnant women in the ANC, a finding supported by an acceptability study involving ISTp in Indonesia [13]. However, this differs slightly from the acceptability study of these same strategies but conducted in the trial setting [12], that noted all cadres of health workers preferred the use of microscopy. Interestingly, it would seem that despite the known limitations of RDTs, ANC providers in this study felt that the benefits of reducing wait times and providing continuity of care outweighed those concerns. This is encouraging given that more sensitive RDTs are now becoming available such that the role of ISTp or a hybrid strategy may be revisited.

Pregnant women were willing to have blood taken if it meant detecting an illness and receiving treatment. Many acknowledged that the finger pricks were painful but that the benefits outweighed the discomfort. This is in line with findings from northern Ghana and Malawi suggesting that women were motivated to endure blood tests in order to find out if they had malaria [11, 22]. Finding out about their health status was a high priority for most women who participated in the discussions. Among women in the intervention group (ISTp), being tested for malaria was a primary motivation to return for subsequent ANC visits, a finding supported by an ISTp acceptability study in Malawi [11]. Healthcare providers also believed that women were willing to accept the blood test, as they trusted the staff and services offered at ANC.

Both providers and pregnant women perceived DP to be an effective anti-malarial with some healthcare providers expressing a preference to DP over SP. Concerns about growing SP resistance led some providers to suggest that shifting to ISTp would help relieve drug pressure. Fewer side effects were reported by women taking DP compared with SP, although the side effects themselves were similar, mainly dizziness, nausea, and headaches. The experience of side effects can affect both acceptability of the drug and adherence to the dosing regimen. That women reported few or mild side effects to DP is encouraging when considering that women need to complete the 2nd and 3rd dose at home. Providers are sometimes skeptical of whether or not women complete doses of medication at home. In Ghana, providers suggested that women would discard the SP tablets if given to take at home [22]. Women expressed dislike for having to take SP on an empty stomach fearing side effects. This finding was documented in a large systematic review on barriers and facilitators to MiP interventions [23] and a study in Mali found that healthcare providers sometimes avoided giving IPTp for this reason [24]. However, across both IPTp and ISTp discussion groups women reported seeing the health benefits of taking anti-malarial medications and accepted what was given to them by the ANC providers. Adherence was only an issue for one woman who was given quinine tablets for malaria treatment and didn’t finish due to side effects.

Key themes to consider on the delivery of ISTp through the ANC platform include: task shifting from the laboratory and pharmacy to ANC nurses, weighing the additional workload for ANC providers against the benefits of providing a ‘one-stop-shop’ at ANC and the sustainability of RDTs and DP considering the cost and supply chain issues. Integration of ISTp into routine ANC elucidated mixed views from providers depending largely on the cadre. Issues with RDT proficiency and adherence to giving the first dose by DOT identified in the quantitative study reflects the reservations by pharmacists about nurses prescribing anti-malarials. This suggests that both pre- and in-service training of ANC providers along with supportive supervision would be required. Specifically, pharmacists could be involved in the training and overseeing of DP provision until confidence in the delivery of the first dose by DOT and adequate instructions for subsequent doses was enhanced.

Front-line ANC nurses were positive about testing women with RDTs and dispensing anti-malarials at ANC as they felt it would improve continuity of care and reduce waiting times. This was supported by the quantitative findings that found testing in the ANC to be efficient as women were promptly given their results and 91% of women who tested positive for malaria were given DP immediately [17]. Although conversely, the study found that only 31% of women received the first dose of DP by DOT, which may reflect the perception by providers that women are motivated to take anti-malarials after they receive a positive RDT result and therefore may not be as concerned about giving the first dose by DOT. In addition, women may refuse DOT due to concerns about taking drugs generally on an empty stomach, a finding consistent with other studies [23, 24]. ANC nurses at control sites (IPTp) were more likely to express concerns about increased workload of using RDTs for malaria screening and felt that sufficient staffing levels needed to be achieved before ISTp could be implemented. That providers at implementation sites did not express concerns about additional workload could mean that either administering RDTs at ANC did not significantly increase their workload or that they perceived the benefits of increased continuity of care to outweigh the additional effort of testing women.

Several providers suggested a hybrid strategy with women tested for malaria at every ANC visit and treated if positive, as per ISTp, with any woman testing negative receiving IPTp-SP to ensure complete protection. This finding was also reported in the acceptability study [12], with providers reluctant to completely let go of a prevention strategy. The proposal for a hybrid strategy may reflect the general lack of confidence in RDT or simply a reluctance to shift paradigms from prevention to treatment.

The RCT of ISTp-DP versus IPTp-SP showed that ISTp was inferior to IPTp and should not replace IPTp at current levels of RDT sensitivity [6]. Since the trial, more sensitive RDTs have become available such that the global community may revisit the ISTp strategy in some settings, for example in areas with decreased malaria transmission when IPTp would no longer be cost effective, or the hybrid strategy, for areas with high levels of SP resistance [25]. The findings of this study enhance the understanding of healthcare provider and pregnant women’s perceptions of test and treat strategies versus prevention for the control of malaria in pregnancy in routine health care settings.

### Strengths and limitations

Focus group discussions with pregnant women relied on historical reports of ANC visits from the current or past pregnancies, although even if some facts are recall-dependent the perspectives and feelings captured are likely to be genuine. The degree to which women feel comfortable sharing their experiences and opinions in a group setting may affect their willingness to give truthful accounts. Social desirability bias may apply to both women and providers interviewed, who may have given answers they feel the interviewer wanted to hear. Findings from this study help explain and support the quantitative results from the parallel study thus strengthening the overall conclusions.

## Conclusions

This study explores healthcare provider and pregnant women perspectives on factors that could affect the implementation of ISTp-DP in routine settings. In general, healthcare providers were accepting of ISTp as a potential alternative to IPTp-SP, a finding reflected in the parallel quantitative study, but many recognized the limitations of ISTp at the current levels of RDT sensitivity. Pregnant women acknowledged the benefits of being tested and treated for malaria. DP was well tolerated by women, who reported minimal side effects. In addition, DP was seen as an effective anti-malarial by healthcare providers, especially with the growing threat of SP resistance. Concerns about RDT and DP availability could represent legitimate barriers to effective implementation. These findings will help inform policy makers when considering the feasibility of implementing ISTp-DP alone or in combination with IPTp-SP.

## Supplementary Information

**Additional file 1.** Healthcare provider IDI topic guide.

**Additional file 2.** Pregnant women FGD topic guide.

## Data Availability

All data generated for this analysis are included in this published article and its additional information files.

## Abbreviations

ANC: Antenatal care
DNMP: Division of National Malaria Programme
DOT: Directly observed therapy
DP: Dihydroartemisinin–piperaquine
FGD: Focus group discussions
IDI: In-depth interview
IPTp: Intermittent preventive treatment in pregnancy
ISTp: Intermittent screening and treatment in pregnancy
LLINs: Long-lasting insecticidal nets
MiP: Malaria in pregnancy
SP: Sulfadoxine–pyrimethamine
RCT: Randomized control trial
RDT: Rapid diagnostic test
