# Supplementary material for: Healthcare provider and pregnant women’s perspectives on the implementation of intermittent screening and treatment with dihydroartemisinin–piperaquine for malaria in pregnancy in western Kenya: a qualitative study
Source: Malar J. 2021 Jun 29;20:291. doi: 10.1186/s12936-021-03826-8 (PMC8243500; doi:10.1186/s12936-021-03826-8)
Supplement: Supplementary file 1 — Additional file 1. Healthcare provider IDI topic guide. [file 12936_2021_3826_MOESM1_ESM.docx]

## In-Depth Interview Theme Guides

## Background characteristics (cover sheet)

Health worker Name:

Interviewer

Health facility

District

Date

Start time

End time

Age

Sex

Highest professional qualification

Tasks perform

Length of time been at HF

## Facility Staff in ANC: Midwives or Nurses

**Section 1. General Information**

What is your role in this health facility?

What does this involve?

Before this role, what was your previous job?

Have you ever worked in the delivery of ANC services anywhere else? Where?

If yes, what did this work involve & for how long did you do this work?

**Section 2. Perceptions of malaria in pregnancy**

What are the problems that might happen if a woman gets malaria while she is pregnant?

What is the best thing to do to avoid these problems?

Are there any other ways to avoid these problems?

Can you tell me what IPTp is? [If they don’t know, then explain that it is intermittent preventive treatment with sulphadoxine-pyrimethamine (SP)] then probe for:

⮚ What it involves

⮚ What it does

Have you had any experience of giving IPTp?

What are your feelings about giving IPTp (good and/or bad)?

**Section 3. Perceptions & experiences of taking blood at ANC**

Have you ever taken blood by finger prick during your ANC duties? If yes, what was it for?

Can you tell me what an “RDT” [*or appropriate term the midwives use*] is?

Have you ever used one of these in the ANC?

What are your feelings about using these in routine ANC?

What do you think the women feel about having finger prick blood tests at ANC?

**Section 4. Prescribing & dispensing drugs at ANC**

What would make you think a pregnant woman at ANC needs an anti-malarial (signs & symptoms)?

⮚ Probe for first and subsequent reasons

If you think a woman at ANC has malaria, what do you do?

⮚ Have you ever prescribed an anti-malarial during your routine ANC duties? If yes, what and why? If no, why not?

If a pregnant woman is prescribed an anti-malarial at this ANC, where do they get the drug?

⮚ Do you think this is the right place? Why?

**Section 5. Perceptions of drugs in pregnancy**

What do you think is the best drug to give a pregnant woman to treat malaria? Why?

What do you think about the use of DHA-piperaquine/quinine/AL for treatment of malaria cases in pregnant women?

What do you think pregnant women feel about being given and using DHA-piperaquine/ quinine/AL for treatment of malaria cases?

What do you think about the use of SP [Fansidar] for IPTp in pregnant women?

What do you think pregnant women feel about being given and using SP [Fansidar] for IPTp?

**Section 6 What do they know about the project**

What do you know about the malaria in pregnancy project that has been running in this ANC?

For us the project meant that when women arrived at ANC, they either get IPTp or they get a blood test and if they are positive they are given treatment for malaria.

- Which of these approaches do you think is better? Why?

If a woman tests positive for malaria there are two treatment choices. She can be given either DHA-piperaquine (trial drug), or quinine (first trimester, per policy) or ACT [AL](second and third trimesters, per policy).

- Which of these do you think is better? Why?

**Section 7: Possible changes to MiP policy in future**

Are you aware of any policy discussions or potential policy changes around prevention and/or management of malaria in pregnancy?

What do you think about the use of RDTs for diagnosis of malaria as an alternative to blood slides?

How would you feel about every pregnant woman being given an RDT test at every ANC visit and if they were positive for malaria they would be given an anti-malarial drug, if they were negative they would not receive any anti-malarial?

How would you feel if the pregnant women attending ANC who tested positive for malaria were given their anti-malaria treatment by the ANC staff? And/or the ANC staff also implemented the RDTs?

⮚ Probe for their feelings on workload, staff levels, management of stocks (anti-malarials, RDTs), record keeping, correct dosage etc

## Laboratory technicians

**Section 1. General information**

What does your position involve?

How long have you been working in this health facility? In this role?

Section 2: Lab tests for ANC

What lab tests do you do for pregnant women?

- Probe for whether this includes malaria tests

What type of test do you do for malaria in pregnant women? Why?

- Probe on when an RDT would be used and when microscopy would be used?

Which type of test do you prefer to do for ANC women? Why?

Is there any reason you would use and alternative method for malaria in ANC women?

**Section 3: Awareness of project**

Are you aware of the malaria in pregnancy project that has been running in this health facility for the last few months? What can you tell me about this project?

What do you feel about this project?

⮚ Probe for views on the different arms, if they know about them

Has this project made any difference to your work? What and why?

How do you feel about nurses doing rapid diagnostic tests for malaria in ANC?

- Is this a good thing? Why? Why not?

**Section 4: Possible changes to MiP policy in future**

Are you aware of any policy discussions or potential policy changes around prevention and/or management of malaria in pregnancy?

What do you think about the use of RDTs for diagnosis of malaria as an alternative to blood slides?

How would you feel about every pregnant woman being given an RDT test at every ANC visit and if they were positive for malaria they would be given an anti-malarial drug, if they were negative they would not receive any anti-malarial?

How would you feel if the pregnant women attending ANC who tested positive for malaria were given their anti-malaria treatment by the ANC staff? And/or the ANC staff also implemented the RDTs?

⮚ Probe for their feelings on workload, staff levels, management of stocks (anti-malarials, RDTs), record keeping, correct dosage etc

## Pharmacist / Dispenser

**Section 1. General information**

1.1 What does your position involve?

1.2 How long have you been working in this district? In this role?

**Section 2. Routine dispensing practices**

What do you routinely dispense to treat malaria in a pregnant woman? Why?

What do you tell pregnant women about how to take these drugs? (Probe for information on dose/duration and if they give any advice on whether to take with food etc.)

What information do you give about the drugs you dispense for malaria? What do you say?

In your experience, are there any differences in the side-effects of DHA-piperaquine and quinine?

- If yes, what are these and what do you tell the women about them?

In your opinion, which is the best drug to give to pregnant women who have been diagnosed with malaria? Why?

?

Do you receive your stocks of quinine, AL and SP from the same source?

Do you ever have problems with stock-outs of quinine, AL and SP?

- If yes, how frequently and what do you do about getting more drugs (can you source from elsewhere?
- While waiting for the new stocks, what do you do instead?

**Section 3: Awareness of project**

Are you aware of the malaria in pregnancy project that has been running in the health facility over the last few months? What can you tell me about this project?

What do you feel about this project?

⮚ Probe for views on the different arms, if they know about them

Has the project had any impact on your work? If yes, in what way?

How do you feel about this?

How would you feel about every pregnant woman requiring an RDT test at every ANC visit and if they were positive for malaria they would be given an anti-malarial drug?

How would you feel if the pregnant women attending ANC who tested positive for malaria were given their anti-malaria treatment by the ANC staff?

**Section 4: Possible changes to MiP policy in future**

Are you aware of any policy discussions or potential policy changes around prevention and/or management of malaria in pregnancy?

What do you think about the use of RDTs for diagnosis of malaria as an alternative to blood slides?

How would you feel about every pregnant woman being given an RDT test at every ANC visit and if they were positive for malaria they would be given an anti-malarial drug, if they were negative they would not receive any anti-malarial?

How would you feel if the pregnant women attending ANC who tested positive for malaria were given their anti-malaria treatment by the ANC staff?

⮚ Probe for their feelings on stock management (anti-malarials, RDTs), record keeping, correct dosage etc.

Please could you explain the drug supply management chain in this district?

Do you ever have problems with stock-outs of SP/quinine/AL in this district?

⮚ If yes, how frequently and what do you do about getting more drugs (can you source from elsewhere)?

⮚ While waiting for the new stocks, what do you do instead?

Do you have problems with stock-outs at health facilities in the district?

⮚ If yes, how frequently and what do you do about managing this?

## District Public Health Nurse / District Medical Officer

**Section 1. General information**

What does your position involve?

How long have you been working in this district? In this role?

**Section 2. Treatment and prevention policy & practices for malaria in pregnancy**

In your opinion, which is the best drug to give to pregnant women who have been diagnosed with malaria? Why?

Do you know what drug is usually given in this district?

In your experience, are there any differences in the side-effects of DHA-piperaquine ,SP, quinine and ACT[AL ]and other anti-malarials. If yes, what are these and what issues do you think they raise?

Do you have experience of seeing patients with side effects to any antimalarials?

⮚ Implications for adherence to full dose, or future prescriptions (by users and providers)

**Section 3: Awareness of project**

Are you aware of the malaria in pregnancy project that has been running in certain ANC facilities in this district for the last few months? What can you tell me about this project?

What do you feel about this project?

⮚ Probe for views on the different arms, if they know about them

What do you feel about ANC staff routinely giving pregnant women DHA-piperaquine to prevent malaria during their pregnancy (IPTp)?

Have you heard any comments from the midwives on giving DHA-piperaquine to pregnant women?

**Section 4: Possible changes to MiP policy in future**

Are you aware of any policy discussions or potential policy changes around prevention and/or management of malaria in pregnancy?

What do you think about the use of RDTs for diagnosis of malaria as an alternative to blood slides?

How would you feel about every pregnant woman being given an RDT test at every ANC visit and if they were positive for malaria they would be given an anti-malarial drug, if they were negative they would not receive any anti-malarial?

How would you feel if the pregnant women attending ANC who tested positive for malaria were given their anti-malaria treatment by the ANC staff? And/or the ANC staff also implemented the RDTs?

⮚ Probe for their feelings on workload, staff levels, management of stocks (anti-malarials, RDTs), record keeping, correct dosage etc
