# Supplementary material for: Healthcare provider and pregnant women’s perspectives on the implementation of intermittent screening and treatment with dihydroartemisinin–piperaquine for malaria in pregnancy in western Kenya: a qualitative study
Source: Malar J. 2021 Jun 29;20:291. doi: 10.1186/s12936-021-03826-8 (PMC8243500; doi:10.1186/s12936-021-03826-8)
Supplement: Supplementary file 2 — Additional file 2. Pregnant women FGD topic guide. [file 12936_2021_3826_MOESM2_ESM.docx]

## Focus Group Discussion Guide: Pregnant women

## Objectives for Discussion Guide Users

Women will be divided into 4 groups based upon their intervention experience during the pilot implementation/feasibility study. These groups will include those who received : 1) ISTp-DP and were RDT negative, 2) ISTp-DP and were RDT positive at least once, 3) IPTp-SP and 4) a heterogeneous group receiving a mix of strategies.

**Malaria and malaria in pregnancy**

What is malaria?

What is malaria in pregnancy?

- Is it different from the malaria they get when not pregnant?

How do they feel when they have malaria?

How do they feel when they are pregnant and have malaria?

- Do they feel ill when they are pregnant? And how does this differ from being ill with malaria when they are pregnant?

What do they do when they are ill with malaria?

What do they do when they are ill with malaria and pregnant?

- If different – reasons why
- Does what they do depend on the cause of the malaria when they are pregnant?
- What are the causes and what do they do?

**Investigate the first visit to ANC: Generally**

To find out why women visited the ANC

- For what reason do they go to ANC?
- For what reason do they not go to ANC?
- How do they feel about the providers (likes, dislikes)?

Were there specific expectations?

**Investigating the first visit to ANC: Linked to study**

To see what they remember about what happened to themselves the first time they went to the ANC in this pregnancy

- What symptoms or conditions trigger the decision to visit a health facility?
- Did they get everything that they thought they needed?
- Any differences to other pregnancies?
- What did they like about what happened that visit ?- reasons
- What did they dislike about what happened that visit ?– reasons

To find out what they know about the medicines that they were given at the first ANC visit

- What drugs were they given? (color, name, number, or size)
- What were the drugs for? (counseled about the drugs)
- How did they take the drugs?
- When did they take the drugs?

To find out whether they had any perceived side effects

What did they feel about these drugs (likes, dislikes)?

- How did they feel after they had taken the drugs?
- If they felt ill – what were the problems
- Are they sure this was due to the drugs? Why?

To find out about any drugs they took home from the ANC clinic

- Did they take them all?
- If they did not take all the drugs, why not (influences)?

To find out what they know about the blood tests they were given

- From which part of the body was the blood taken and how many times?
- What were the tests for?
- How do they feel about these blood tests (likes, dislikes)?

To see if they can remember what other women got

- Did everyone get the same?
- If not, why, what, how did they feel?

**Investigating repeat visits to ANC: Linked to study**

To find out what they remember about what happened on return visits to ANC

- Did they get everything that they thought they needed?
- Any differences to their previous pregnancies?
- Any differences to their first visit during this pregnancy?
- What did they like about what happened during these visits? – reasons
- What did they dislike about what happened during these visits? – reasons
- Did any of these experiences motivate them to come back, or put them off returning?

**Investigating fever during pregnancy**

- To find out if they had a fever during their current pregnancy
- To find out what they did (when, where, why?)

**Additional questions, concerns or comments**

- Before closing the discussion, check if there are any further questions or comments to add
